# Supplementary material for: Miktoarm Star Copolymers Prepared by Transformation from Enhanced Spin Capturing Polymerization to Nitroxide-Mediated Polymerization (ESCP-Ŧ-NMP) toward Nanomaterials
Source: Nanomaterials (Basel). 2021 Sep 14;11(9):2392. doi: 10.3390/nano11092392 (PMC8467092; doi:10.3390/nano11092392)
Supplement: Supplementary file 1 [file nanomaterials-11-02392-s001.zip › nanomaterials-1355637-supplementary.pdf]

nanomaterials

MDPI

Supplementary material

Miktoarm Star Copolymers Prepared by Transformation from Enhanced Spin Capturing Polymerization to Nitroxide-Mediated Polymerization (ESCP-*T*-NMP) toward Nanomaterials

Tzu-Yao Lin [1], Cheng-Wei Tu [2], Junko Aimi [3], Yu-Wen Huang [1], Tongsai Jamnongkan [4], Han-Yu Hsueh [5], Kun-Yi Andrew Lin [6,*] and Chih-Feng Huang [1,*]

[1] Department of Chemical Engineering, i-Center for Advanced Science and Technology (iCAST), National Chung Hsing University, Taichung 40227, Taiwan; s05310141@gmail.com (T.-Y.L.); enjoy1995427@gmail.com (Y.-W.H.)
[2] Industrial Technology Research Institute, Chutung, Hsinchu 31057, Taiwan; CWTu@itri.org.tw
[3] Research Center for Functional Materials, National Institute for Materials Science, 1-2-1 Sengen, Tsukuba, Ibaraki 305-0047, Japan; AIMI.Junko@nims.go.jp
[4] Department of Fundamental Science and Physical Education, Faculty of Science at Sriracha, Kasetsart University, Chonburi 20230, Thailand; jamnongkan.t@ku.ac.th
[5] Department of Materials Science and Engineering, National Chung Hsing University, Taichung 40227, Taiwan; hyhsueh@nchu.edu.tw
[6] Department of Environmental Engineering, Innovation and Development Center of Sustainable Agriculture, Research Center of Sustainable Energy and Nanotechnology, iCAST, National Chung Hsing University, Taichung 40227, Taiwan
* Correspondence: linky@nchu.edu.tw (K.-Y.A.L.), HuangCF@dragon.nchu.edu.tw (C.-F.H.)

Captions.

Scheme S1. Synthesis of (1Z,1'Z)-1,1'-(1,4-phenylene)-bis(*N*-*tert*-butylmethanimine oxide) nitrone (i.e., PBBN) (where, t-BuHAA: *N*-(*tert*-butyl)hydroxylamine acetate).

Figure S1. (**A**) FT-IR (4000–400 cm^{-1}) and (**B**) ^1H NMR (400 MHz, $CDCl_3$, *: solvent peaks) spectra of PBBN nitrone.

Figure S2. FT-IR spectra of μ-star copolymers before and after hydrolysis.

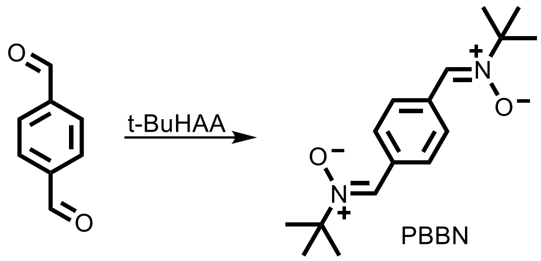

Scheme S1. Synthesis of (1Z,1'Z)-1,1'-(1,4-phenylene)-bis(N-tert-butylmethanimine oxide) nitrone (i.e., PBBN) (where, t-BuHAA: N-(tert-butyl)hydroxylamine acetate).

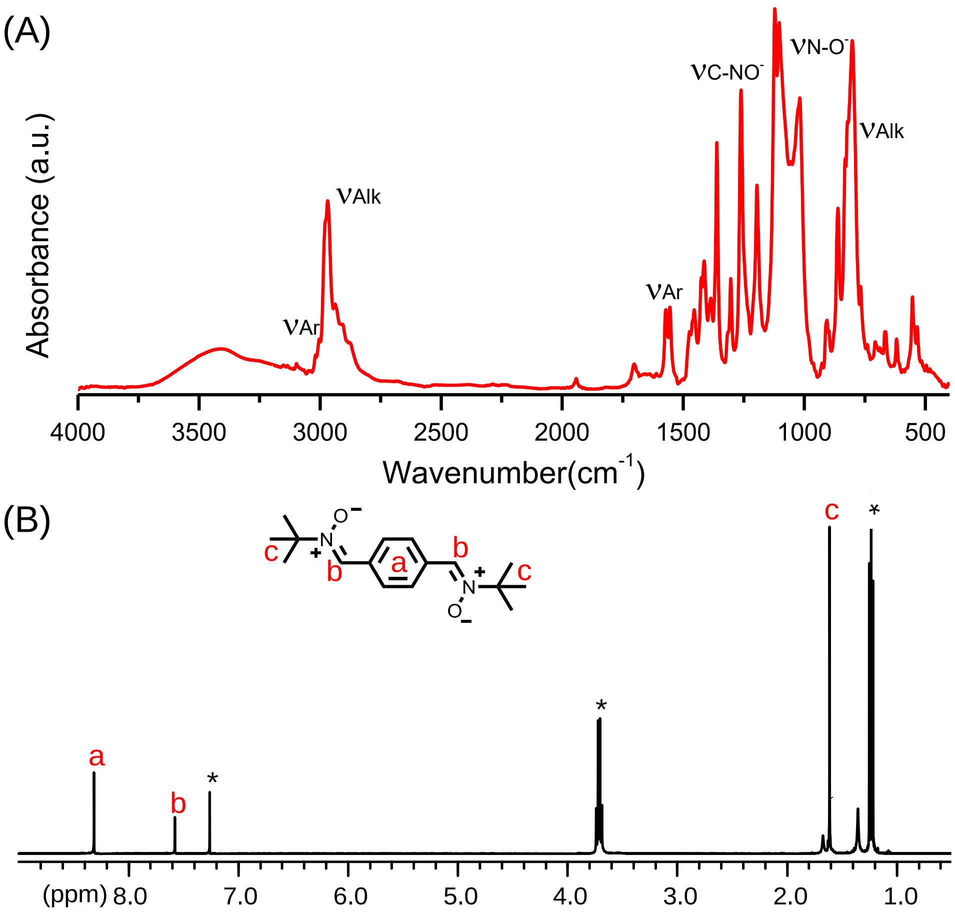

Figure S1. (**A**) FT-IR (4000–400 cm^{-1}) and (**B**) ^1H NMR (400 MHz, $CDCl_3$, *: solvent peaks) spectra of PBBN nitrone.

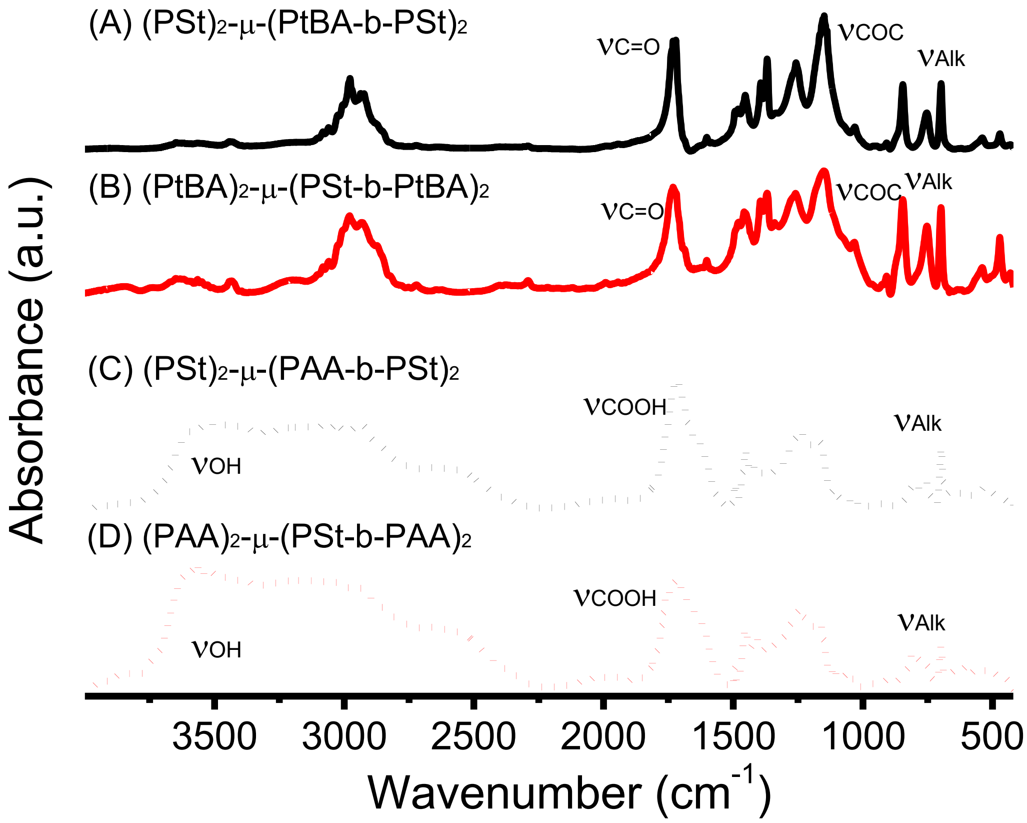

Figure S2. FT-IR spectra of μ-star copolymers before and after hydrolysis.
